# Survival nomogram for patients with bone metastatic renal cell carcinoma: A population-based study

**DOI:** 10.1590/S1677-5538.IBJU.2020.0195

**Published:** 2021-02-03

**Authors:** Keyi Wang, Zonglin Wu, Guangchun Wang, Heng Shi, Jinbo Xie, Lei Yin, Tianyuan Xu, Weipu Mao, Bo Peng

**Affiliations:** 1 Tongji University School of Medicine People's Hospital of Putuo District Shanghai China Department of Urology, People's Hospital of Putuo District, School of Medicine, Tongji University, Shanghai; 2 Tongji University School of Medicine Shanghai Tenth People's Hospital Shanghai China Department of Urology, Shanghai Tenth People's Hospital, School of Medicine, Tongji University, Shanghai; 3 Southeast University Affiliated Zhongda Hospital Department of Urology Nanjing China Department of Urology, Affiliated Zhongda Hospital of Southeast University, Nanjing, China

**Keywords:** Carcinoma, Renal Cell, Nomograms, SEER Program, Survival

## Abstract

**Purpose:**

Increased attention has been focused on the survival of renal cell carcinoma (RCC) patients with bone metastasis. This study proposed to establish and evaluate a nomogram for predicting the overall survival (OS) and cancer-specific survival (CSS) of RCC patients with bone metastasis.

**Materials and Methods:**

RCC patients with bone metastasis between 2010 and 2015 were captured from the surveillance, epidemiology and end results (SEER) database. Univariate and multivariate cox regressions were performed to assess the effects of clinical variables on OS and CSS. The nomogram based on the Cox hazards regression model was developed. Concordance index (C-index) and calibration curve were performed to evaluate the accuracy of nomogram models, receiver operating characteristic (ROC) curves and decision curve analysis (DCA) were conducted to assess the predict performance.

**Results:**

A total of 2.471 eligible patients were enrolled in this study. The patients were assigned to primary (n=1.672) and validation (n=799) cohorts randomly. The 1-, 2-, and 3-year OS and CSS nomogram models were constructed based on age at diagnosis, sex, marital status, pathological grade, T-stage, N-stage, brain/liver/lung metastasis, surgery, radiotherapy and chemotherapy. The c for OS and CSS prediction was 0.730 (95% confidence interval [CI]: 0.719-0.741) and 0.714 (95%CI:0.702-0.726). The calibration curves showed significant agreement between nomogram models and actual observations. ROC and DCA indicated nomograms had better predict performance.

**Conclusions:**

The nomograms for predicting prognosis provided an accurate prediction of OS and CSS in RCC patients with bone metastasis, and contributed clinicians to optimize individualized treatment plans.

## INTRODUCTION

Renal cell carcinoma (RCC) arises from abnormal differentiation of renal tubular epithelial cells (1). About 2-3% of malignant diseases in adults are RCC and clear cell RCC (ccRCC), that accounts for about 82-90% of RCC, is the most common type (2). Nearly 20-30% of RCC patients were metastatic RCC (mRCC) at the time of diagnosis and commonly spread to bones (1, 3). Approximately 85% of RCC patients with bone metastasis presented skeletal-related events (SRE) such as pathological fractures (4).

Approximately 209.000 new RCC patients were diagnosed every year worldwide (5) and the 5-year survival rate is close to 45% for these patients (4). However, the survival time after metastasis is about 12 months for mRCC patients (6). There were studies found that the prognosis of RCC patients with bone metastasis is closely related to age, TNM stage, other organ metastasis, whether receive targeted treatment (7, 8). However, these predictions have not been validated effectively due to the rarity of the disease and there is still a lack of a predictive model that calculates different variables simultaneously. In recent years, nomogram has been regarded as a reliable model for predicting tumor prognosis considering the unique calculation method (9, 10). This provides a new method for prognostic analysis of RCC patients with bone metastasis. Our work will establish the nomogram predicting the prognosis of RCC patients with bone metastasis to assist clinicians in developing individualized treatment plans.

This study evaluated data from the surveillance, epidemiology and end results (SEER) database (11), further investigated the factors affecting the prognosis of RCC patients with bone metastasis, and then applied the obtained results to the construction of nomograms. The nomograms were established and verified by the Cox regression results from the patient's information of SEER database. This helped determine the relationship between different clinic factors and patient's overall survival (OS) and cancer––specific survival (CSS).

## MATERIALS AND METHODS

### Patients selection

Clinical data of RCC patients with bone metastasis from 2010 to 2016 obtained from the SEER database from National Cancer Institute through SEER*Stat software (version 8.3.5; SEER 18 Regs Custom Data (with additional treatment fields), Nov 2018 Sub (1975-2016 varying) database). As one of the largest public cancer datasets, it covers 28% of the U.S. population (12). Additionally, the metastasis information related to liver, lung, bone and brain was published since 2010. We identified 100.813 patients with RCC based on the “Primary Site-labeled” variable, between January 1, 2010 and December 31, 2016.

**Figure 1:**
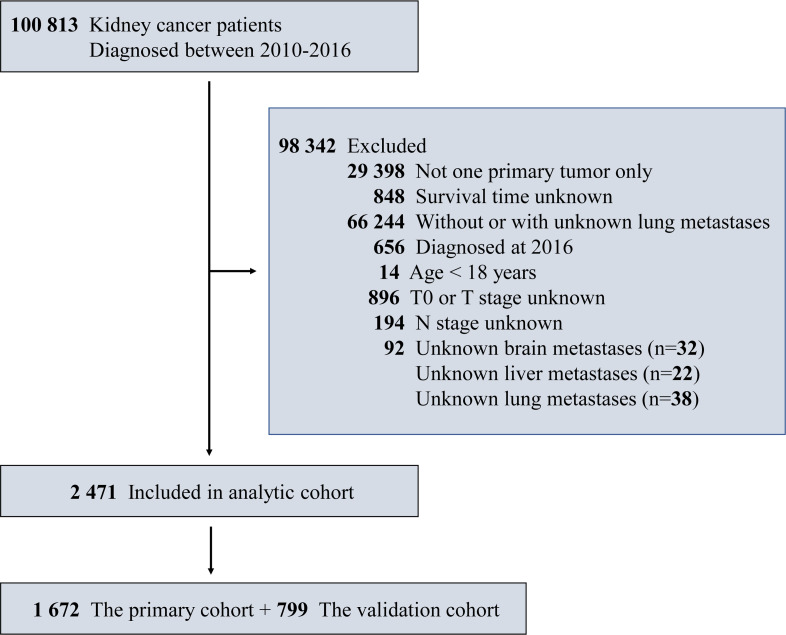
Study design flowchart of specific patient screening process.

The exclusion criteria for patients adopted in our study were: a) more than one primary tumor; b) unknown survival time; c) without or with unknown lung metastasis; d) diagnosed at 2016; e) age at diagnosis under 18 years; f) T0 or T-stage unknown; g) N-stage unknown; h) unknown brain/liver/lung metastasis. Cases diagnosed after January 1, 2016 were excluded for the purpose of obtaining follow––up observations more than one year for all patients. In this study, the entire cohort included 2.471 eligible patients and the eligible patients were randomly assigned to the primary and validation cohorts. The detailed study design was shown in Figure-1. This study protocol was approved by the Biomedical Ethics Committee of the Tenth Hospital in Shanghai (IRB number: SHSY-IEC-KY-4.0/18-68/01).

### Study variables

The following clinical information for each patient were obtained from the SEER database: the year of diagnosis, age at diagnosis, sex, marital status, pathological grade, T-stage (AJCC, 7th ed.), N–stage (AJCC, 7th ed.), brain/liver/lung metastasis, surgery, radiotherapy and chemotherapy. The age at diagnosis was classified into the following groups: <40, 40-59, 60-79 and ≥80. Unmarried group of marital status included divorced/separated, widowed and single patients. The pathological grade was detailed divided into well differentiated, moderately differentiated, poorly differentiated, undifferentiated and unknown. OS time means the patient's survival time from diagnosis to any cause leading to death or the date on which data were censored. The study only analyzed cancer-specific survival times and excluded deaths associated with other causes when CSS was the endpoint. The cut-off point of our study was set on December 31, 2016.

### Statistical Analysis

Kaplan-Meier curve and log-rank test were performed to investigate the OS and CSS of bone metastatic RCC and the difference analysis. Univariate and multivariate regression analysis was used to evaluate the prognostic factors in RCC patients with bone metastasis. Receiver operating characteristic (ROC) curves and decision curve analysis (DCA) were conducted to assess the predict performance of nanograms and TNM-stage (13). The statistical software package for social science software (version 20.0; SPSS, Chicago, USA) was applied for all statistical analyses.

The Cox proportional hazards results in the entire cohort were the basis for the construction and verification of nomograms. R software version 3.5.1 (http://www.R-project.org) was performed for establishing nomograms. The package of R applied in this study were “rms” and “rmda” (13). Concordance index (C-index) and calibration curve were performed to evaluate the performance and accuracy of nomo-grams. The C-index value ranges from 0.50 to 1.00 and shows a positive correlation with the predicted performance of the model. It indicates the models accompanied with perfect discrimination ability when the value is 1.00. And when the calibration curve is applied to a perfectly calibrated model, the prediction will fall on the diagonal 45° in the figure. The results were considered statistically significant as P-value <0.05 (two-sided).

## RESULTS

### Patients baseline characteristics

There were 2.471 eligible RCC patients with bone metastasis enrolled in the statistical analysis. All eligible patients were divided into the primary cohort (n=1.672) and the validation cohort (n=799) randomly. For age at diagnosis, there were 1.234 (49.9%) patient's age ranged from 60 to 79. In the sex groups, there were 1.707 (68.8%) male patients. The marital status grouping result of patients showed 1.387 (56.1%) patients were married. As for N-stage, the N0-group accounted for 64.3% (1.589) of all patients. Most patients had no brain (2.199; 89.0%) and liver (2.001; 81.0%) metastasis. However, there were 1.244 (50.3%) patients with lung metastasis. The treatment protocol of patients included surgery (912; 36.9%), radiotherapy (1.327; 53.7%) and chemotherapy (1.376; 55.7%). The clinical characteristics of the patients are detailed shown in Table-1.

**Table 1:** Baseline demographic and clinical characteristics with bone metastatic kidney cancer patients in our study.

Characteristic		Total No. (%)	The primary cohort	The validation cohort
			No. (%)	No. (%)
Total		2471	1672	799
**Year of diagnosis**				
	2010	357 (14.4)	257 (15.4)	100 (12.5)
	2011	394 (15.9)	267 (16.0)	127 (15.9)
	2012	398 (16.1)	293 (17.5)	105 (13.1)
	2013	417 (16.9)	265 (15.8)	152 (19.0)
	2014	438 (17.7)	288 (17.2)	150 (18.8)
	2015	467 (18.9)	302 (18.1)	165 (20.7)
**Age at diagnosis**				
	< 40	132 (5.3)	81 (4.8)	51 (6.4)
	40-59	823 (33.3)	543 (32.5)	280 (35.0)
	60-79	1234 (49.9)	860 (51.4)	374 (46.8)
	≥ 80	282 (11.4)	188 (11.2)	94 (11.8)
**Sex**				
	Male	1707 (69.1)	1151 (68.8)	556 (69.6)
	Female	764 (30.9)	521 (31.2)	243 (30.4)
**Marital status**				
	Married	1387 (56.1)	951 (56.9)	436 (54.6)
	Unmarried	1084 (43.9)	721 (43.1)	363 (45.4)
**Grade**				
	Grade I	31 (1.3)	19 (1.1)	12 (1.5)
	Grade II	198 (8.0)	137 (8.2)	61 (7.6)
	Grade III	485 (19.6)	330 (19.7)	155 (19.4)
	Grade IV	309 (12.5)	216 (12.9)	93 (11.6)
	Unknown	1448 (58.6)	970 (58.0)	478 (59.8)
**T stage**				
	T1	759 (30.7)	500 (29.9)	259 (32.4)
	T2	548 (22.2)	357 (21.4)	191 (23.9)
	T3	913 (36.9)	636 (38.0)	277 (34.7)
	T4	251 (10.2)	179 (10.7)	72 (9.0)
**N stage**				
	N0	1589 (64.3)	1072 (64.1)	517 (64.7)
	N1	498 (20.2)	343 (20.5)	155 (19.4)
	N2	384 (15.5)	257 (15.4)	127 (15.9)
**With** brain **metastases**				
	No	2199 (89.0)	1487 (88.9)	712 (89.1)
	Yes	272 (11.0)	185 (11.1)	87 (10.9)
**With liver metastases**				
	No	2001 (81.0)	1373 (82.1)	628 (78.6)
	Yes	470 (19.0)	299 (17.9)	171 (21.4)
**With lung metastases**				
	No	1227 (49.7)	840 (50.2)	387 (48.4)
	Yes	1244 (50.3)	832 (49.8)	412 (51.6)
**Surgery**				
	No	1559 (63.1)	1034 (61.8)	525 (65.7)
	Yes	912 (36.9)	638 (38.2)	274 (34.2)
**Radiotherapy**				
	No	1144 (46.3)	768 (45.9)	376 (47.1)
	Yes	1327 (53.7)	904 (54.1)	423 (52.9)
**Chemotherapy**				
	No	1095 (44.3)	743 (44.4)	352 (44.1)
	Yes	1376 (55.7)	929 (55.6)	447 (55.9)

**Grade I** = Well differentiated; **Grade II** = Moderately differentiated; **Grade III** = Poorly differentiated; **Grade IV** = Undifferentiated.

Percentages may not total 100 because of rounding.

### Cox regression analyses for prognostic factors of OS and CSS

Univariate and multivariate regression analysis were performed to investigate the independent prognostic factors for OS and CSS of RCC patients with bone metastasis. The clinical variables under statistical analysis were as follows: age at diagnosis, sex, marital status, pathological grade, T-stage, N-stage, brain/liver/lung metastasis, surgery, radiotherapy and chemotherapy. Table-2 shows the detailed results.

**Table 2:** Univariate and multivariate analysis of overall survival (OS) and cancer-specific survival (CSS) rates.

Characteristic		OS				CSS			
		Univariate analysis		Multivariate analysisa		Univariate analysis		Multivariate analysisb	
		Hazard Ratio (95% CI)	P value	Hazard Ratio (95% CI)	P value	Hazard Ratio (95% CI)	P value	Hazard Ratio (95% CI)	P value
**Age at diagnosis**									
	< 40	Reference		Reference		Reference		Reference	
	40-59	0.89 (0.73-1.09)	0.259	0.84 (0.69-1.03)	0.098	0.87 (0.71-1.08)	0.202	0.83 (0.67-1.03)	0.084
	60-79	1.07 (0.88-1.31)	0.470	1.01 (0.83-1.23)	0.932	1.02 (0.83-1.25)	0.856	0.97 (0.79-1.19)	0.736
	≥ 80	1.71 (1.37-2.14)	<0.001	1.25 (1.00-1.58)	0.051	1.48 (1.16-1.87)	0.001	1.13 (0.89-1.45)	0.314
**Sex**									
	Male	Reference				Reference			
	Female	1.08 (0.99-1.19)	0.089			1.08 (0.97-1.19)	0.159		
**Marital status**									
	Married	Reference		Reference		Reference		Reference	
	Unmarried	1.25 (1.15-1.37)	<0.001	1.12 (1.02-1.22)	0.014	1.22 (1.11-1.34)	<0.001	1.11 (1.01-1.22)	0.039
**Grade**									
	Grade I	Reference		Reference		Reference		Reference	
	Grade II	0.81 (0.51-1.28)	0.357	0.83 (0.52-1.32)	0.438	0.82 (0.51-1.33)	0.423	0.85 (0.53-1.39)	0.525
	Grade III	1.15 (0.74-1.78)	0.534	1.30 (0.83-2.02)	0.250	1.12 (0.71-1.78)	0.630	1.25 (0.78-2.00)	0.347
	Grade IV	1.40 (0.90-2.19)	0.136	1.65 (1.05-2.60)	0.030	1.41 (0.89-2.26)	0.147	1.61 (1.00-2.60)	0.049
	Unknown	2.00 (1.30-3.07)	0.002	1.11 (0.72-1.71)	0.645	1.88 (1.19-2.96)	0.006	1.06 (0.67-1.67)	0.819
**T stage**									
	T1	Reference		Reference		Reference		Reference	
	T2	1.09 (0.97-1.23)	0.153	1.00 (0.88-1.13)	0.981	1.18 (1.03-1.34)	0.014	1.06 (0.93-1.21)	0.394
	T3	0.97 (0.87-1.07)	0.510	1.16 (1.03-1.31)	0.013	1.07 (0.95-1.20)	0.265	1.27 (1.11-1.44)	<0.001
	T4	1.61 (1.39-1.87)	<0.001	1.21 (1.04-1.42)	0.017	1.76 (1.50-2.07)	<0.001	1.31 (1.11-1.56)	0.002
**N stage**									
	NO	Reference		Reference		Reference		Reference	
	N1	1.60 (1.44-1.79)	<0.001	1.35 (1.20-1.50)	<0.001	1.58 ()1.40-1.77	<0.001	1.31 (1.16-1.47)	<0.001
	N2	1.71 ()1.52-1.93	<0.001	1.52 (1.34-1.72)	<0.001	1.75 (1.54-1.99)	<0.001	1.50 (1.31-1.71)	<0.001
**With brain metastases**									
	No	Reference		Reference		Reference		Reference	
	Yes	1.56 (1.37-1.78)	<0.001	1.30 (1.14-1.72)	<0.001	1.64 (1.42-1.88)	<0.001	1.34 (1.16-1.55)	<0.001
**With liver metastases**									
	No	Reference		Reference		Reference		Reference	
	Yes	1.99 (1.79-2.22)	<0.001	1.52 (1.36-1.70)	<0.001	1.95 (1.74-2.19)	<0.001	1.45 (1.29-1.64)	<0.001
**With lung metastases**									
	No	Reference		Reference		Reference		Reference	
	Yes	1.71 (1.57-1.87)	<0.001	1.43 (1.30-1.58)	<0.001	1.76 (1.60-1.93)	<0.001	1.43 (1.29-1.59)	<0.001
**Surgery**									
	No	Reference		Reference		Reference		Reference	
	Yes	0.38 (0.35-0.42)	<.001	0.36 (0.31-0.41)	<0.001	0.40 (0.36-0.44)	<0.001	0.36 (0.31-0.41)	<0.001
**Radiotherapy**									
	No	Reference		Reference		Reference		Reference	
	Yes	0.80 (0.73-0.87)	<0.001	-	0.195	0.85 (0.77-0.93)	0.001	-	0.566
**Chemotherapy**									
	No	Reference		Reference		Reference		Reference	
	Yes	0.68 (0.63-0.74)	<0.001	0.53 (0.49-0.59)	<0.001	0.75 (0.68-0.83)	<0.001	0.58 (0.52-0.64)	<0.001

**OS** = Overall survival; **CSS** = Cancer-specific survival; **Grade I** = Well differentiated; **Grade II** = Moderately differentiated; **Grade III** = Poorly differentiated; Grade IV, Undifferentiated.

^a^ Model was adjusted by age at diagnosis, marital status, Grade, T stage, N stage, and metastases pattern.

^b^ Model was adjusted by age at diagnosis, marital status, Grade, T stage, N stage, and metastases pattern.

In our study, there were several variables impacted the prognosis of patients. From the results of univariate analysis, we found that age at diagnosis, marital status, pathological grade, T-stage, N-stage, brain/liver/lung metastasis, surgery, radiotherapy and chemotherapy were associated with OS and CSS. Unmarried patients had the poor OS (Hazard Ratio [HR]=1.25; 95% CI:1.15-1.37; P <0.001) and CSS (HR=1.22; 95% CI:1.11-1.34; P <0.001). For OS, the RCC patients with brain (HR=1.56; 95% CI:1.37-1.78; P <0.001), liver (HR=1.99; 95% CI:1.79-2.22; P <0.001) and lung (HR=1.71; 95% CI:1.57-1.87; P <0.001) metastasis were accompanied by worse survival compared with the reference. Simultaneously, these patients had worse CSS (P <0.001). For patients accepted different treatment, surgery (HR=0.38; 95% CI:0.35-0.42; P <0.001), radiotherapy (HR=0.80; 95% CI:0.73-0.87; P <0.001) and chemotherapy (HR=0.68; 95% CI:0.63-0.74; P <0.001) were benefic for the OS. Statistical results also indicate that treatment was beneficial to the patient's CSS.

Results from multivariate analysis indicated the conclusions consisted with the univariate analysis. These clinic variables included age at diagnosis, marital status, T-stage, N-stage, brain/liver/lung metastasis, surgery and chemotherapy had impacted the OS and CSS of patients. The poor OS (HR=1.23; 95% CI:1.12-1.34; P <0.001) and CSS (HR=1.20; 95% CI:1.10-1.32; P <0.001) occurred in the unmarried patients. The worse OS was found in patients with brain (HR=1.38; 95% CI:1.21-1.58; P <0.001), liver (HR=1.60; 95% CI:1.43-1.79; P <0.001) and lung (HR=1.42; 95% CI:1.29-1.55; P <0.001) metastasis. Patients with surgery (HR=0.36; 95% CI:0.31-0.41; P <0.001) and chemotherapy (HR=0.53 95% CI:0.49-0.59; P <0.001) treatments gained a better OS. And the CSS was worse than reference in those patients from the statistic results.

### Construction and verification of Nomograms

**Figure 2:**
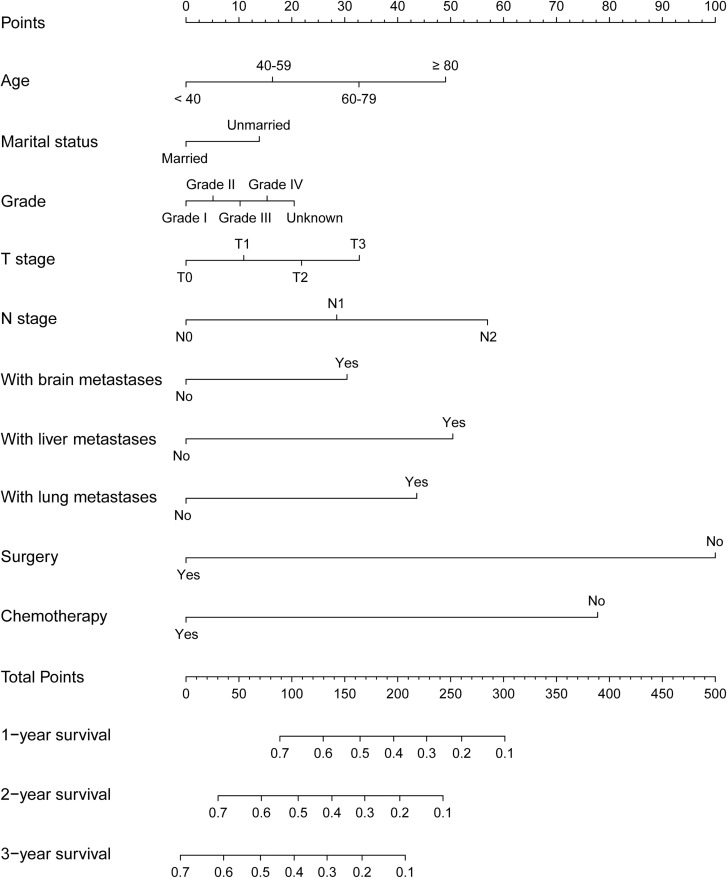
The 1-, 2-, and 3-year nomogram model for overall survival (OS) for patients with bone metastatic renal cell carcinoma (RCC).

**Figure 3:**
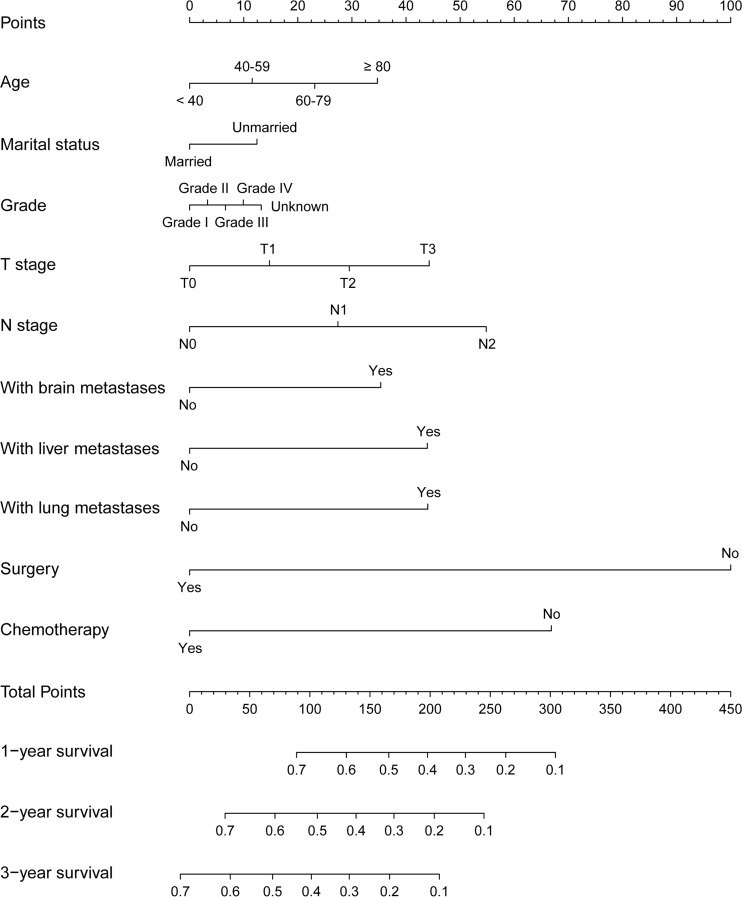
The 1-, 2-, and 3-year nomogram model for cancer-specific survival (CSS) for patients with bone metastatic RCC.

The variables based on regression analysis for the entire cohort were included in the construction of nomograms. The included clinic factors were age at diagnosis, sex, marital status, pathological grade, T-stage, N-stage, brain/liver/lung metastasis, surgery and chemotherapy. According to the multivariate cox regression results, the 1-, 2-, and 3-year OS and CSS nomogram models were established. Figure-2 showed the 1-, 2-, and 3-year OS nomogram developed by the Cox proportional hazards results. And the 1-, 2-, and 3-year CSS can be founded in Figure-3. The length of the line corresponding to each variable in nomograms represents the effect of clinical variables on patient's survival outcomes and each subtype of the variable corresponds to a point on the “point” scale. The corresponding “total points” can be obtained by adding the scores associated with each variable and the projection of “total points” can be used to estimate the probability of 1-, 2-, and 3-year OS and CSS.

**Figure 4:**
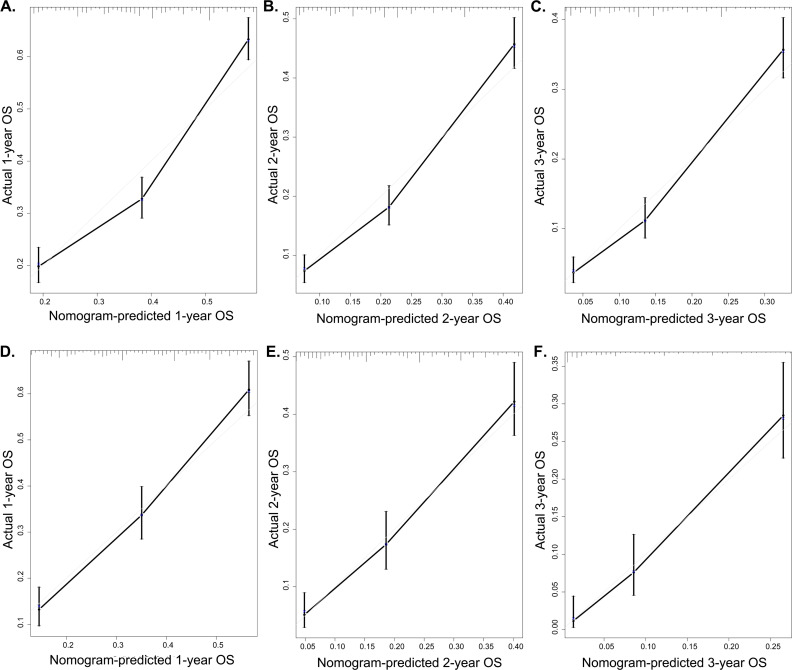
Calibration plot of the 1-, 2-, and 3-year OS nomogram. The calibration curves of the 1-year (A), 2-yaer (B), and 3-year (C) nomogram model for OS in the primary cohort respectively; The calibration curves of the 1-year (D), 2-year (E), and 3-year (F) nomogram model for OS in the validation cohort respectively.

**Figure 5:**
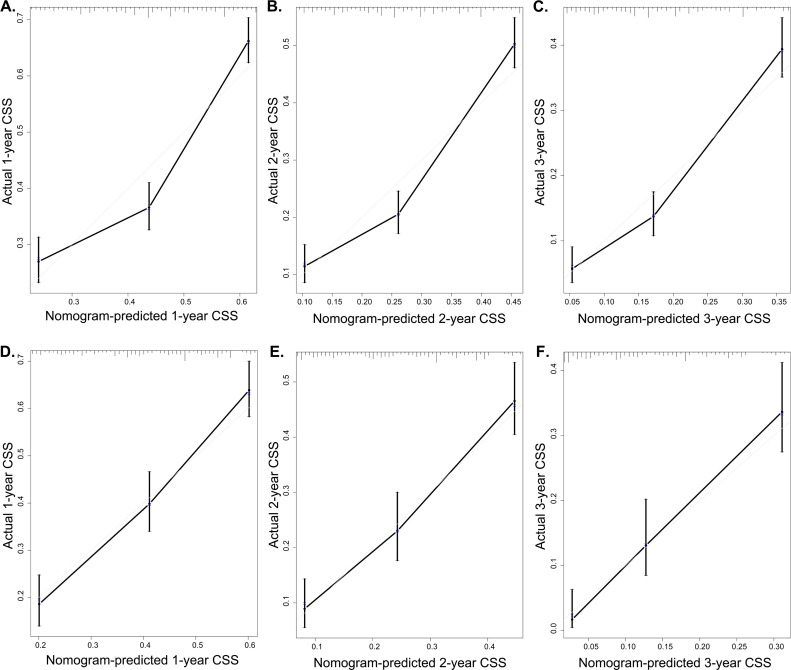
Calibration plot of the 1-, 2-, and 3-year CSS nomogram. The calibration curves of the 1-year (A), 2-year (B), and 3-year (C) nomogram model for CSS in the primary cohort respectively; The calibration curves of the 1-year (D), 2-year (E), and 3-year (F) nomogram model for CSS in the validation cohort respectively.

In addition, the C-index was conducted to further assess the predictive performance of the models. For the entire cohort, the C-index values were 0.730 (95% CI: 0.719-0.741) of OS and 0.714 (95% CI:0.702-0.726) of CSS. Simultaneously, calibration curve as a calibration tool was developed to evaluate the accuracy of nomogram models based on the primary and validation cohort's results. The evaluations were performed using a bootstrap with 1000 resamples. The validation of OS nomogram is showed in Figure-4. The calibration of the 1-, 2-, and 3-year CSS nomogram are depicted in Figure-5. The results showed that there was a good agreement between the predictions of the nomograms and the actual observations in the primary cohort and the verification cohort.

**Figure 6:**
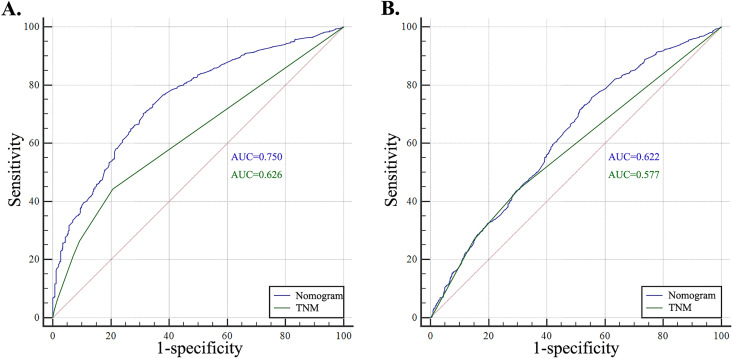
Receiver operating characteristic (ROC) analysis based on nomograms and TNM-stage. (A) The ROC analysis of OS; (B) The ROC analysis of CSS.

**Figure 7:**
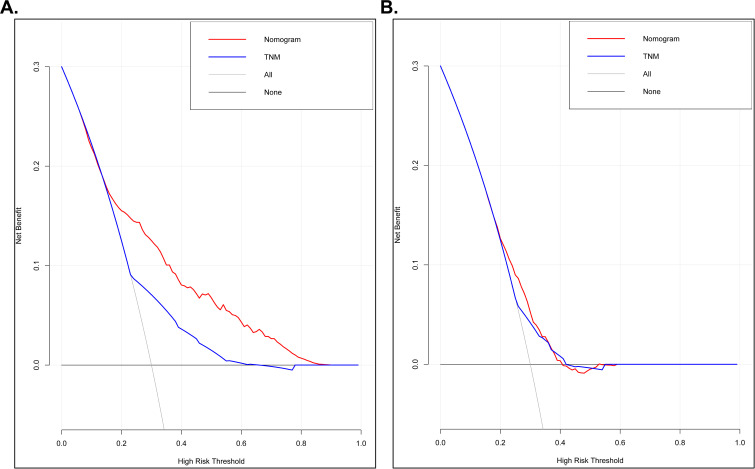
Decision curve analysis (DCA) based on nomograms and TNM-stage. (A) DCA of OS for patients with bone metastatic RCC; (B) DCA of CSS for patients with bone metastatic RCC.

The ROC analysis was conducted based on nanograms and TNM-stage and results showed nanograms hold a better predict performance than TNM-stage. The area under curve (AUC) of nano-grams were 0.756 (OS) and 0.618 (CSS) respectively, which can be found in Figure-6. DCA curve was used to assess whether nanograms would help with clinical treatment strategies in Figure-7. In our study, when the threshold probability varied from 0 to 1, nanograms achieved the most net benefit compared with TNM-stage according to the DCA. It was found that nanograms can better predict the OS and CSS in RCC patients with bone metastasis than TNM-stage.

## DISCUSSION

In this study, we firstly established prognostic nomograms for OS and CSS of RCC patients with bone metastasis. According to the results of Cox regression, we could predict the 1-, 2-, and 3-year OS and CSS by the construction of nomograms. The nomograms we developed in this study contained the following clinical variables: age at diagnosis, marital status, T-stage, N-stage, brain/liver/lung metastasis, surgery and chemotherapy. Frontline clinicians can optimize personalized treatment plans based on the detailed situation of the related clinical characteristics for patients. This will assist the RCC patients with bone metastasis to obtain better survival benefits and prolong the survival time.

There had been many studies found that age, sex and marital status were independent prognostic factors for patients of various cancer (14). We further explored the mechanism of their effect on patient's survival. For patients of different cancer types, the immune system will weaken with the increase of age and this will help tumor deterioration further reduce patient's survival (15). In practice, there was evidence proved that C-reactive protein (CRP) could predict the prognosis of mRCC patients (16). The prognosis of various cancer patients of different genders may be related to inconsistent hormone levels in the body (17), for example the testosterone, estrogen and progesterone level's change will cause specific cancer (18). This confirmed that sex as a prognostic factor for OS and CSS. As for marital status, the impact on survival was related with the personal emotional support, high quality of care and financial support (19). And the widowed patients had worse OS and CSS compared with the married patients. Those would be the reasons for our conclusion consistent with previous studies (20).

Tumor-related pathological characteristics have also been found to be correlated with the prognosis of cancer patients, such as pathological grade, T-stage, N-stage and multi-organ metastasis (21). Cancer stem cells were one of the focuses of current studies (22). The pathological grade of the tumor was positively correlated with the stemness of the cancer cells (22). High-grade tumors were often accompanied by a high degree of malignancy and strong invasiveness, which has an adverse impact on the prognosis of patients (23). Simultaneously, increased expression of CD133 and nestin in high-grade tumor tissues lead to an increase of cell atypia and reduced effectiveness of medical treatment (23). In our study, the pathological grade of tumors as an independent prognostic factor influenced the survival of RCC patients with bone metastasis. This was consistent with conclusions from previous studies, such as bladder cancer, prostate cancer and others (24, 25).

TNM-stage is currently the most universal tumor staging system in the world. Tumor's TNM––stage is defined based on the results of laboratory tests and postoperative pathological examination (26). It as an independent prognostic factor for cancer patients and has been confirmed by many studies (21). Clinicians would determine the TNM stage based on individualized situation of tumor (T), node (N) and metastasis (M) in cancer patients. And the T-stage represents the condition of the primary tumor, which is determined based on the tumor volume and the surrounding tissue involvement. The N-stage illustrates the involvement of regional lymph nodes. As for the M-stage, it means whether the tumor tissue metasta-sized. For cancer patients with different TNM stages, higher stage means complicated medical treatment and short survival time (27). Those explained the reasons for that T-stage, N-stage, brain metastasis, liver metastasis and lung metastasis were independent influencing factors for patient's prognosis in our study.

Medical treatment for patients with bone metastasis included surgery, radiotherapy and chemotherapy in this study. Reducing tumor burden through various medical methods can benefit the survival of patients. Studies have found that surgery can prolong the survival time of RCC patients with bone metastasis and even the patients with advanced metastasis could still obtain better survival benefits from surgery (28). Radiotherapy and chemotherapy are also the main medical treatments for various cancer. However, radiotherapy is mainly used for palliative treatment of RCC patients due to the insensitivity, which is also consistent with our research results (29). Recently, tyrosine kinase inhibitors such as sunitinib and sorafenib have been used in the clinical treatment of advanced RCC and achieved surprising results (29). Chemotherapy has benefited from the invention of these biological agents and achieved certainly clinical effects on a variety of cancers.

Although the impact of these independent prognostic factors has been reported, there is a lack of a predictive model that can incorporate these factors into the analysis simultaneously. In recent years, nomograms had been applied to types of cancer as an extremely effective prediction model for predicting patient's survival (30). In this study, the ROC and DCA results indicated nomogram have better predictive performance compared to TNM-stage. Nomograms combined mathematical models and biological results and considered the different clinical characteristics and pathological variables of the cancer patients comprehensively, then graphically showed the possibility of clinical results. As our results showed nanograms perform better than individual indicators. The nomograms had higher accuracy in predicting the patient's prognosis than existing prediction models (31). Clinicians could make intuitive quantitative predictions of patient's survival based on the nomo-grams, and this would further guide the formulation of treatment plans.

In this study, we established the nomograms for OS and CSS of RCC patients with bone metastasis in order to assist to the medical care. Additionally, the results of the C-index and calibration curves both indicated that nomograms had excellent predictive performance. Nevertheless, there were some limitations in this study. Firstly, the SEER database was a retrospective data set and the data may be biased due to manual recording reasons. Secondly, the clinical data we obtained from the SEER database were incomplete, for example the information about comorbidities was not acquired. Thirdly, the analyzed data only included patient's information in the United States between 2010 and 2016, that could not represent other regions. Therefore, it is necessary to conduct multicenter prospective clinical trials to verify the accuracy of the nomograms.

## CONCLUSIONS

In this study, we first established prognostic nomograms for RCC patients with bone metastasis based on the SEER database. Simultaneously, the 1-, 2-, and 3-year OS and CSS nomogram model's accuracy were evaluated by C-index and calibration curves. Nomograms constructed in our study will contribute to the treatment of RCC patients with bone metastasis.

## ABBREVIATIONS

RCC: renal cell carcinoma
OS: overall survival
CSS: cancer-specific survival
SEER: surveillance, epidemiology and end results
ROC: receiver operating characteristic
DCA: decision curve analysis
mRCC: metastatic renal cell carcinoma
SER: skeletal-related events
C-index: Harrell's concordance index
IRB: Institutional Review Board
AJCC: American Joint Committee on Cancer
HR: hazard ratio
CI: confidence interval
AUC: area under curve
